# Protective efficacy of a novel simian adenovirus vaccine against lethal MERS-CoV challenge in a transgenic human DPP4 mouse model

**DOI:** 10.1038/s41541-017-0029-1

**Published:** 2017-10-16

**Authors:** Vincent J. Munster, Daniel Wells, Teresa Lambe, Daniel Wright, Robert J. Fischer, Trenton Bushmaker, Greg Saturday, Neeltje van Doremalen, Sarah C. Gilbert, Emmie de Wit, George M. Warimwe

**Affiliations:** 1 Laboratory of Virology, Division of Intramural Research, National Institute of Allergy and Infectious Diseases, National Institutes of Health, Rocky Mountain Laboratories, Hamilton, MT USA; 2 The Jenner Institute, University of Oxford, Oxford, UK; 3 Rocky Mountain Veterinary Branch, Division of Intramural Research, National Institute of Allergy and Infectious Diseases, National Institutes of Health, Hamilton, MT USA; 4 The Pirbright Institute, Woking, UK; 5 KEMRI-Wellcome Trust Research Programme, Kilifi, Kenya

## Abstract

Middle East respiratory syndrome coronavirus (MERS-CoV) is a novel zoonotic virus that causes severe respiratory disease in humans with a case fatality rate close to 40%, but for which no vaccines are available. Here, we evaluated the utility of ChAdOx1, a promising replication-deficient simian adenovirus vaccine vector platform with an established safety profile in humans and dromedary camels, for MERS-CoV vaccine development. Using a transgenic lethal BALB/c MERS-CoV mouse model we showed that single dose intranasal or intramuscular immunisation with ChAdOx1 MERS, encoding full-length MERS-CoV Spike glycoprotein, is highly immunogenic and confers protection against lethal viral challenge. Immunogenicity and efficacy were comparable between immunisation routes. Together these data provide support for further evaluation of ChAdOx1 MERS vaccine in humans and dromedary camels, the animal reservoir of infection.

## Introduction

Middle East respiratory syndrome coronavirus (MERS-CoV) first emerged in Saudi Arabia in mid-2012^1^ and as of March 2017 more than 1917 laboratory-confirmed cases with >650 related deaths had been officially reported to the World Health Organization (WHO). Most infections (>80%) have been geographically linked to Saudi Arabia, but travel-related cases have occurred in Europe, Asia and Africa.^1^ Dromedary camels are susceptible to MERS-CoV infections and appear to be the main reservoir of virus. However, human-to-human transmission underlies rapid spread of MERS-CoV within hospital settings.^2^ High seroprevalence of antibodies against MERS-CoV has been reported in dromedary camels in the Middle East and various countries in Africa indicating widespread MERS-CoV circulation.^3^

No licensed vaccines or treatments are currently available for MERS-CoV infections. Ongoing disease control strategies have so far relied on minimising contact with dromedary camels, observing standard infection control measures to limit nosocomial transmission, contact tracing and quarantine. Addressing this unmet need for MERS-CoV interventions has been prioritised by the WHO for urgent action^4^ and could most rapidly be achieved through a one health approach in which products are co-developed for use in humans (to prevent disease) and camels (to limit virus shedding and block subsequent transmission to humans). Leveraging vaccine technology platforms with an established safety profile in both these target species would allow relatively rapid progression through the product development pipeline.

Many vaccine platforms have been safely evaluated in humans but vaccine research and development for camelid infections is neglected. Among the most promising human vaccine platforms are replication-deficient simian adenovirus vectors (ChAd), which boasts a very good safety and immunogenicity profile in humans as demonstrated in clinical trials against a wide range of indications including malaria, HIV, tuberculosis, influenza, hepatitis C, Ebola and others.^5^ One ChAd vector, termed ChAdOx1,^6^ has undergone testing in dromedary camels, showing excellent safety and immunogenicity when encoding Rift Valley Fever viral glycoproteins.^7^ We recently made a vaccine construct, ChAdOx1 MERS,^8^ encoding the full-length MERS-CoV spike glycoprotein (GenBank accession number KJ650098.1) targeted by protective neutralising antibodies.^9^ The spike glycoprotein transgene sequence was inserted in the ChAdOx1 E1 region, included a human tissue plasminogen activator signal sequence in the N-terminus, and its expression was under the control of the human major immediate early cytomegalovirus promoter including intron A. ChAdOx1 MERS was shown to elicit high-titre MERS-CoV neutralising antibodies and a robust CD8+ T cell response against the spike glycoprotein.^8^ Here, to determine ChAdOx1 MERS vaccine efficacy we utilised a recently developed transgenic lethal BALB/c mouse model (van Doremalen et al., submitted) expressing the human dipeptidyl peptidase (hDPP4) gene in the Rosa26 locus, which renders mice susceptible to MERS-CoV infection.^10^ Infection with MERS-CoV in the hDPP4 mouse model is uniformly lethal with a dose of 10^3^ TCID50 or higher. MERS-CoV infection is characterised by an initial respiratory phase and a secondary encephalitic phase, similar to what has been described previously.^11^

## Results

**Fig. 1:**
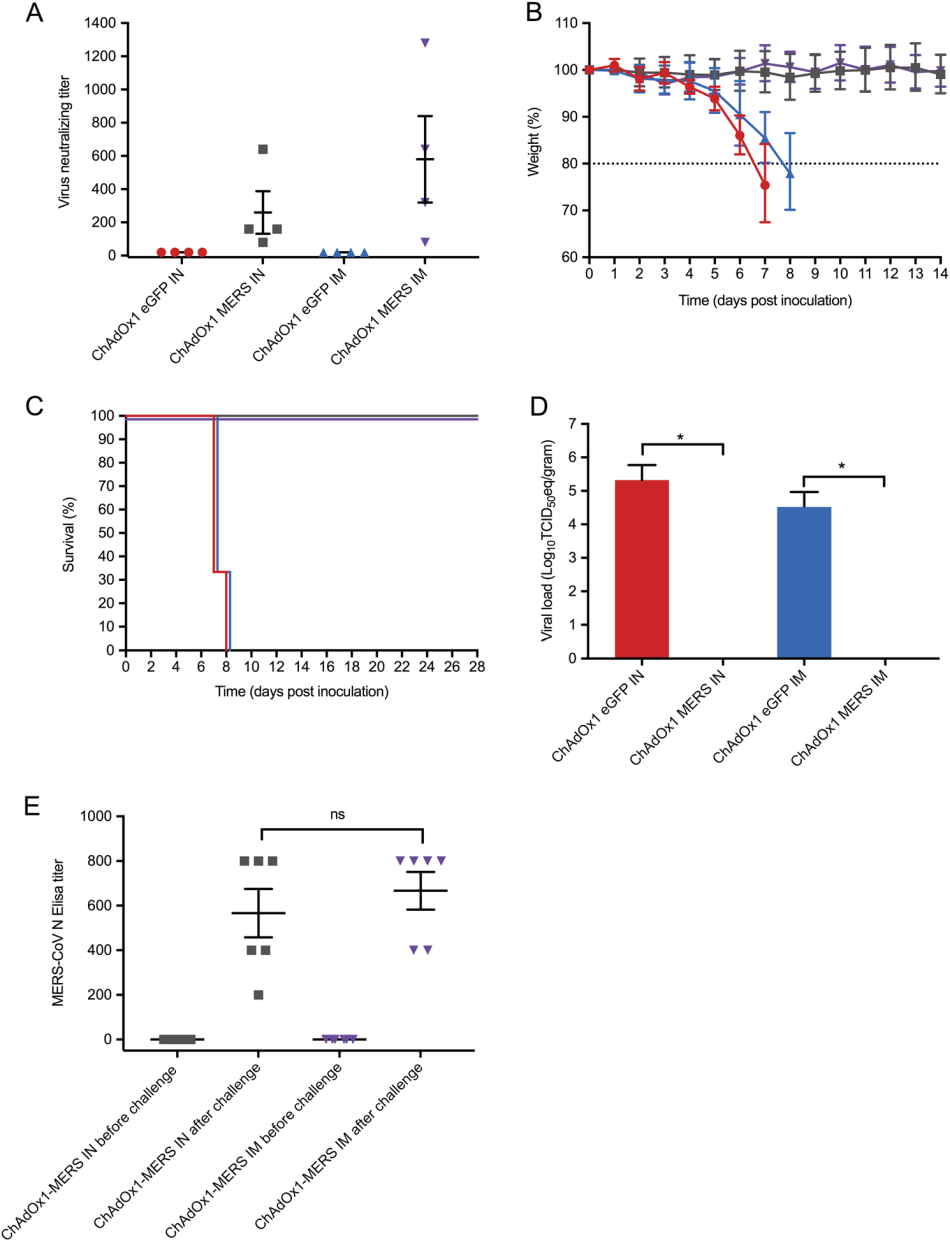
Protective Efficacy of ChAdOx1 MERS vaccine. Groups of 10 mice were vaccinated with 10^8^ Infectious Units (IU) ChAdOx1 eGFP or ChAdOx1 MERS via the intranasal or intramuscular route, blood samples were collected before vaccination, and before challenge at 28 days post vaccination. hDPP4 mice were challenged intranasally with 10^4^ TCID_50_ MERS-CoV (strain HCoV-EMC2012), four animals of each group were euthanized at 3 days post inoculation for serological, virological and histopathological analyses. All analyses were performed in duplicate. **a** Neutralising antibody titre of hDPP4 mouse serum samples against MERS-CoV strain HCoV-EMC/2012 after vaccination (*n* = 4 per group). **b** Weight loss after intranasal challenge with 10^4^ TCID_50_ MERS-CoV, (*n* = 6 per group). **c** Survival curves of the vaccinated groups (*n* = 6 per group). **d** Mean ± SD of MERS-CoV viral loads in the lower respiratory tract of vaccinated hDPP4 mice at 3 dpi (*n* =  4 per group). **e** Nucleocapsid ELISA responses (*n* =  6 per group). All experimental procedures were performed as previously described.^16^ Red = ChAdOx1 eGFP intranasally vaccinated animals; Grey = ChAdOx1 MERS intranasally vaccinated animals; Blue = ChAdOx1 eGFP intramuscularly vaccinated animals; Purple = ChAdOx1 MERS intramuscularly vaccinated animals

ChAdOx1 MERS vaccination elicited neutralising antibodies with no statistically significant difference detected between immunisation routes (Mann Whitney *U* test, *p* = 0.49) (Fig. 1a). No MERS-CoV neutralising antibody response was observed among the ChAdOx1 vaccine encoding enhanced green fluorescent protein (ChAdOx1 eGFP) vaccinees.

To evaluate vaccine efficacy animals were challenged intranasally at 28 days post-vaccination with 10^4^ TCID_50_ of the HCoV-EMC/2012 MERS-CoV strain in a total volume of 25 µl and observed daily for signs of disease. Euthanasia was indicated at >20% loss of initial body weight. At 3 days post-inoculation (dpi), four animals from each group were euthanized and lungs collected for analyses. The remaining six animals per group were sacrificed 28 dpi, or when they reached the humane endpoint criteria. These group sizes were sufficient to allow detection of 100% efficacy in the ChAdOx1 MERS group compared to controls with 90% power using a two-sample comparison of proportions at an alpha of 5% as determined in Stata^®^ v12 statistical software. ChAdOx1 eGFP vaccinees developed signs of disease, including loss of body weight, ruffled fur and lethargy (Fig. 1b). Weight loss begun 3 dpi and at 7–8 dpi all mice in the ChAdOx1 eGFP groups either succumbed to infection or reached the predefined euthanasia criteria (Fig. 1c). No signs of disease or significant loss of body weight were observed in mice vaccinated with ChAdOx1 MERS (Fig. 1b, c).

**Fig. 2:**
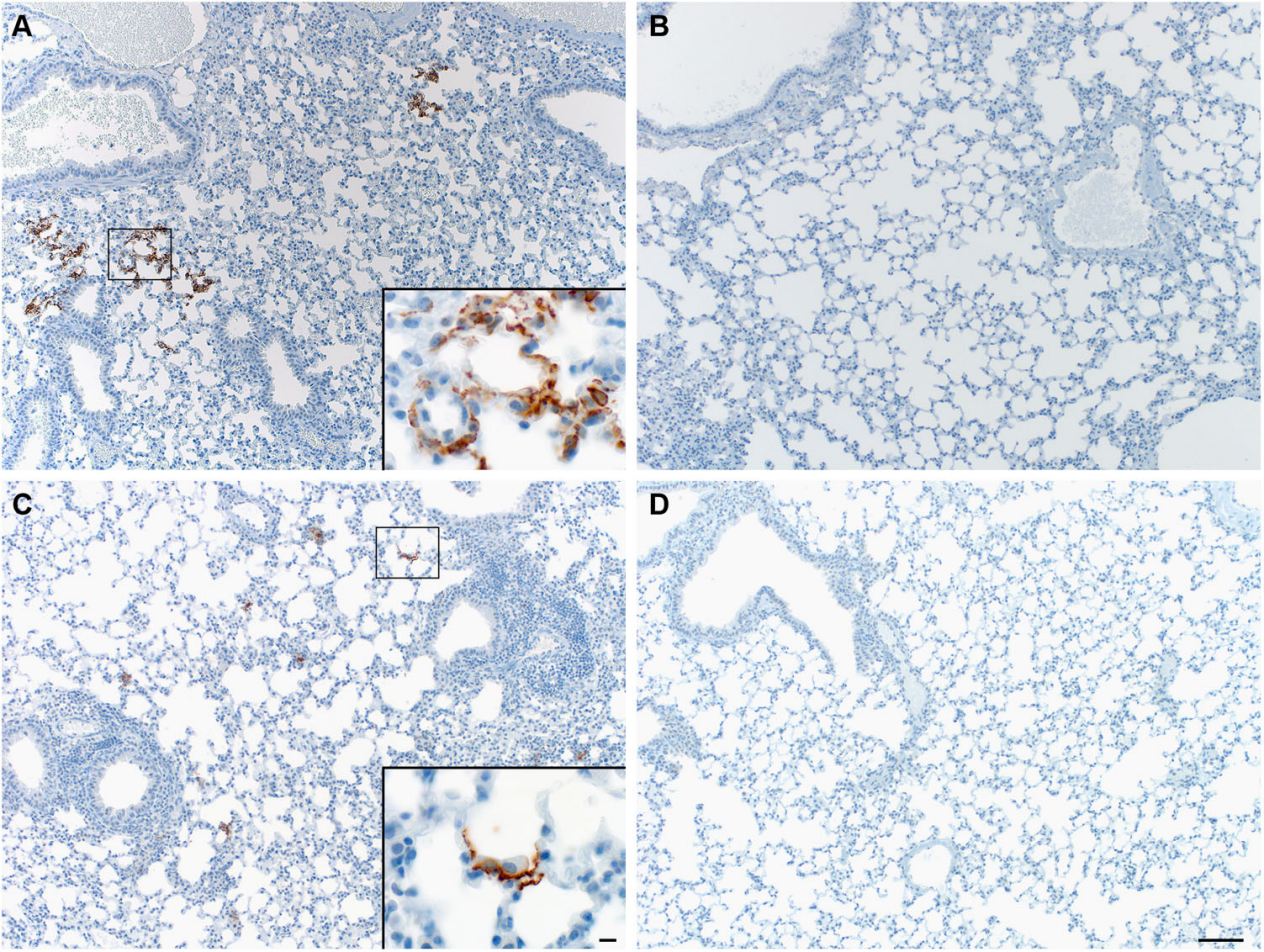
Immunohistochemistry staining for MERS-CoV antigen in the lower respiratory tract of vaccinated hDPP4 mice. hDPP4 mouse tissues were evaluated for pathology and the presence of viral antigen as described previously.^16^ Briefly, tissues were fixed in 10% neutral-buffered formalin for 7 days and paraffin-embedded. Tissue sections were stained with hematoxylin and eosin (H&E). An in-house produced rabbit polyclonal antiserum against HCoV-EMC/2012 (1:1000) was used as a primary antibody for the detection of viral antigen. Grading of histopathology and immunohistochemistry was done blinded by a board-certified veterinary pathologist. Lung tissues are shown at 100 and 1000× (insert) magnification. ChAdOx1 eGFP intranasally (**a**) and intramuscularly (**c**) vaccinated animals show multifocal scattered positivity in the lungs. The inserts display MERS-CoV antigen within the Type I and II pneumocytes. ChAdOx1 MERS intranasally (**b**) and intramuscularly (**d**) vaccinated animals showed no MERS-CoV antigen positivity

The presence of MERS-CoV RNA in the lungs and brains was analysed by qRT-PCR on mice (*n* = 4/group) sacrificed at 3 dpi. High viral loads were found in the lower respiratory tract but not the brains of the ChAdOx1 eGFP-vaccinated mice (intranasal 10^5.32^ TCID_50_ eq/g tissue, 95% confidence interval (CI): 10^2.28^–10^5.77^, intramuscular 10^4.51^ TCID_50_ eq/g tissue, 95% CI: 10^3.1^–10^4.96^). No viral RNA was detected in any of the ChAdOx1 MERS vaccinated mice (Fig. 1d). Immunohistochemistry staining for MERS-CoV in lung tissue showed abundance of antigen in the ChAdOx1 eGFP-vaccinated mice, but not the ChAdOx1-MERS vaccines (Fig. 2). MERS-CoV replication was only observed in the type I and type II pneumocytes (Fig. 2 inserts) but not in any of the other respiratory cells such as endothelial cells, bronchiolar epithelium or macrophages of the ChAdOx1 eGFP control animals. No MERS-CoV antigen was observed at 3 dpi, in the brains of any of the mice. However, as our emphasis was on assessing vaccine efficacy against the respiratory phase of disease, no brain samples later than the peak of virus replication in the respiratory tract (3 dpi) were collected.

To address whether or not the single dose of ChAdOx1 MERS vaccine truly resulted in sterile immunity, we analysed the pre and post challenge sera with a MERS-CoV nucleoprotein ELISA. Irrespective of the route of immunisation, relatively low levels of IgG antibodies against nucleoprotein were detected (Fig. 1e), indicating that the animals were likely briefly infected during the first 1–2 days after inoculation but that this did not result in morbidity and mortality. No significant difference in the MERS-CoV nucleoprotein response was detected between the vaccinated groups (Mann Whitney *U* test, *p* = 0.6970; Fig. 1e).

## Discussion

Together these data provide support for further evaluation of ChAdOx1 MERS vaccine in humans and dromedary camels. This should be relatively straightforward given the established safety profile of the ChAdOx1 platform in humans^5^ and dromedary camels.^7^ A deployable human MERS-CoV vaccine will need to be safe and efficacious in at-risk populations, including healthcare workers, camel herders and those with comorbidities as highlighted in the ongoing WHO-led consultation on an ideal target product profile for MERS-CoV vaccines. However, a major gap remains in the understanding of key immune mechanisms responsible for protection from disease; whilst MERS-CoV infections elicit high titre neutralising antibody in camels, these do not appear sufficient to provide long-term protection against re-infection.^12–14^ Identification of immune correlates of protection against MERS-CoV in humans and camels will allow cost-effective disease surveillance and vaccine monitoring.

In summary, we have demonstrated the utility of the ChAdOx1 platform for MERS-CoV vaccine development in a lethal mouse model. The excellent immunogenicity and efficacy observed here will underpin future evaluations of ChAdOx1 MERS in dromedary camels and humans.

## Methods

hDPP4 mice were randomly assigned to intranasal or intramuscular vaccination with 10^8^ infectious units of either a control ChAdOx1 eGFP or the ChAdOx1 MERS vaccine. The experiment was performed blinded and the experimenters had no knowledge of group allocation of the individual mice and the analysed samples. Sera were obtained before vaccination and 28 days post vaccination and post challenge analysed with a virus neutralisation assay with HCoV-EMC/2012 MERS-CoV or ELISA as described previously.^12,15^

Approval of animal experiments was obtained from the Institutional Animal Care and Use Committee of the Rocky Mountain Laboratories. The performance of experiments was done following the guidelines of the Association for Assessment and Accreditation of Laboratory Animal Care, International (AAALAC) by certified staff in an AAALAC-approved facility, following the guidelines and basic principles in the United States Public Health Service Policy on Humane Care and Use of Laboratory Animals and the Guide for the Care and Use of Laboratory Animals. Work with infectious MERS-CoV strains under BSL3 conditions was approved by the Institutional Biosafety Committee (IBC). Inactivation and removal of samples from high containment was performed according to IBC-approved standards.

### Data availability

All data generated or analysed during this study are included in this published article.
